# Sarcomatoid Carcinoma: A Clinicopathological Dichotomy

**DOI:** 10.7759/cureus.53565

**Published:** 2024-02-04

**Authors:** Rhitam Ghosal, Durba Roychowdhury, Rudra Prasad Chatterjee, Mehebuba Sultana, Sangeeta Sinha

**Affiliations:** 1 Oral and Maxillofacial Pathology, Guru Nanak Institute of Dental Sciences and Research, Kolkata, IND

**Keywords:** fnac, spindle cell, pet-ct, immunohistochemistry, pan-ck, vimentin, spindle cell carcinoma, biphasic, sarcomatoid carcinoma

## Abstract

Spindle cell carcinoma (SpCC) or sarcomatoid carcinoma is an uncommon biphasic malignant neoplasm occurring mainly in the upper aerodigestive tract. It has spindled or pleomorphic tumor cells simulating a true sarcoma with epithelial origin. WHO recognized this tumor as an aggressive variant of squamous cell carcinoma and further designated it as spindle cell carcinoma. This neoplasm is known for its propensity of recurrence and metastasis reinforcing the importance of its proper diagnosis. In our case report, we talk about sarcomatoid carcinoma involving the oral cavity having a high metastasizing rate according to histopathological and immunohistochemical features.

## Introduction

Spindle cell carcinoma (SpCC) is an uncommon variant of squamous cell carcinoma having unusual clinicopathological characteristics, which is recognized by the World Health Organization (WHO) as sarcomatoid squamous cell carcinoma [1]. This tumor has various other names, i.e., polypoid squamous cell carcinoma, pseudosarcoma, collision tumor, and pleomorphic carcinoma [2]. According to Palla et al., SpCC has similar demographics when compared to conventional squamous cell carcinoma showing male predominance and a larger age group with predominantly fifth to sixth decades of life. Habits related to the usage of tobacco, alcohol consumption, trauma, and history of radiation therapy, have a strong correlation with the occurrence of this tumor [1]. SpCC occurs mainly in the larynx, upper aerodigestive tract, salivary glands, skin, breast, urogenital tract, and gastrointestinal tract. It is rarely noted in the oral cavity, pharynx, and sinonasal tract [1,3]. Furthermore, according to their article, Palla et al. mention that SpCC comprises less than 1% of all tumors in the oral cavity. Intraorally most commonly affected sites are the alveolar mucosa, tongue, buccal mucosa, and lower lip [1]. A clinicopathologic review of 103 cases of Indian patients from a tertiary referral cancer center by Viswanathan et al. showed site distribution to be 63.1% for oral cavity tumors in the case of sarcomatoid carcinoma [4]. Clinically this growth usually appears as a polypoidal or pedunculated mass which may cause obstructive symptoms. These oral and/or pharyngeal tumors may be tender or non-tender with a bleeding tendency on provocation along with dysphagia [3]. It is very aggressive in nature with a high recurrence rate and metastases which can be a challenge to the clinician. In a clinicopathological and immunohistochemical study of 40 cases by Sarma et al., it was identified that the recurrence rate was found to be 71.4% and the metastasis rate was 21.4% [5]. SpCC consists of elongated spindle cells that resemble a sarcoma and are considered a form of poorly differentiated squamous cell carcinoma (SCC) [6-7]. Immunohistochemistry and histopathology are of the utmost importance and crucial for the proper diagnosis of the tumor [6].

## Case presentation

A 62-year-old male patient reported to the Department of Oral & Maxillofacial Pathology, Guru Nanak Institute of Dental Sciences & Research, Kolkata, India, with the chief complaint of a nodular growth present on the right mandibular gingivobuccal sulcus in relation to the molar teeth since last 1 month. According to the patient, initially, this growth was a small pea-sized over the right lower gums area measuring around 1 cm x 1 cm, a month back, then gradually the lesion increased in size to almost four times its original size due to self-traumatization thus making the lesion 4 cm x 3 cm in dimensions. He was on medication for hypothyroidism 50 and anxiety for 1 year with the habit of tobacco consumption (khaini) 5-10 times daily for the last three decades.

**Figure 1:**
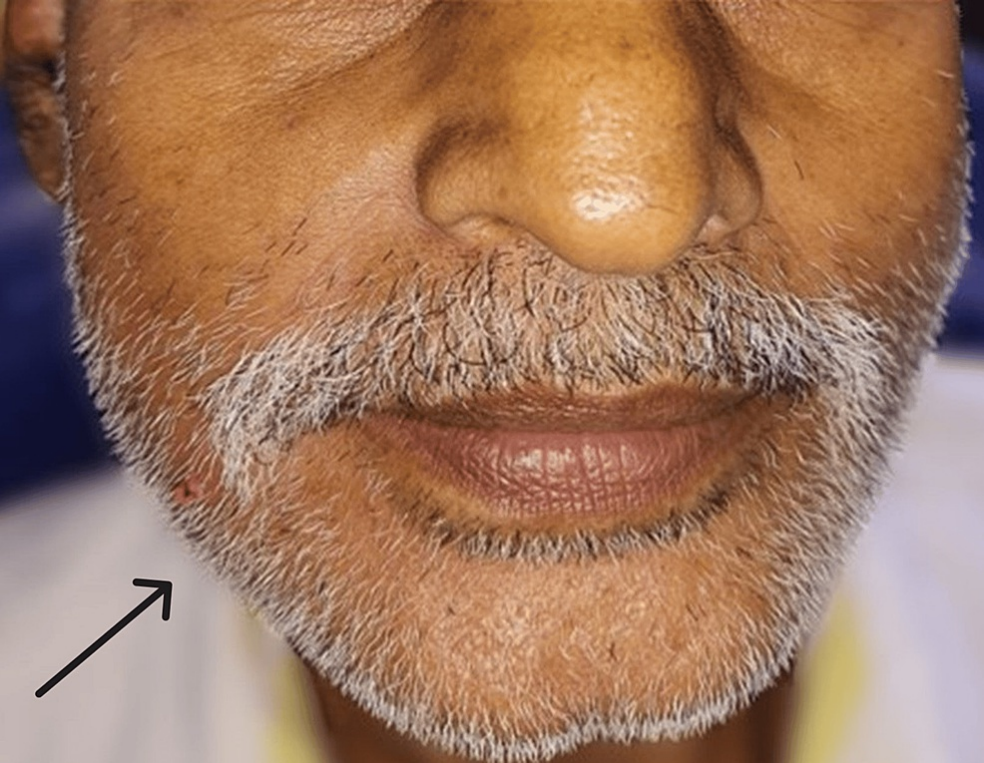
Extraoral photograph of the patient showing a mild, diffuse swelling over the right lower third of the face, near the angle of the mandible.

On extra-oral examination, a mild, diffuse, non-tender swelling was noted over the right lower third of the face, near angle of the mandible (Figure 1). No signs of paresthesia or other abnormality were detected.

**Figure 2:**
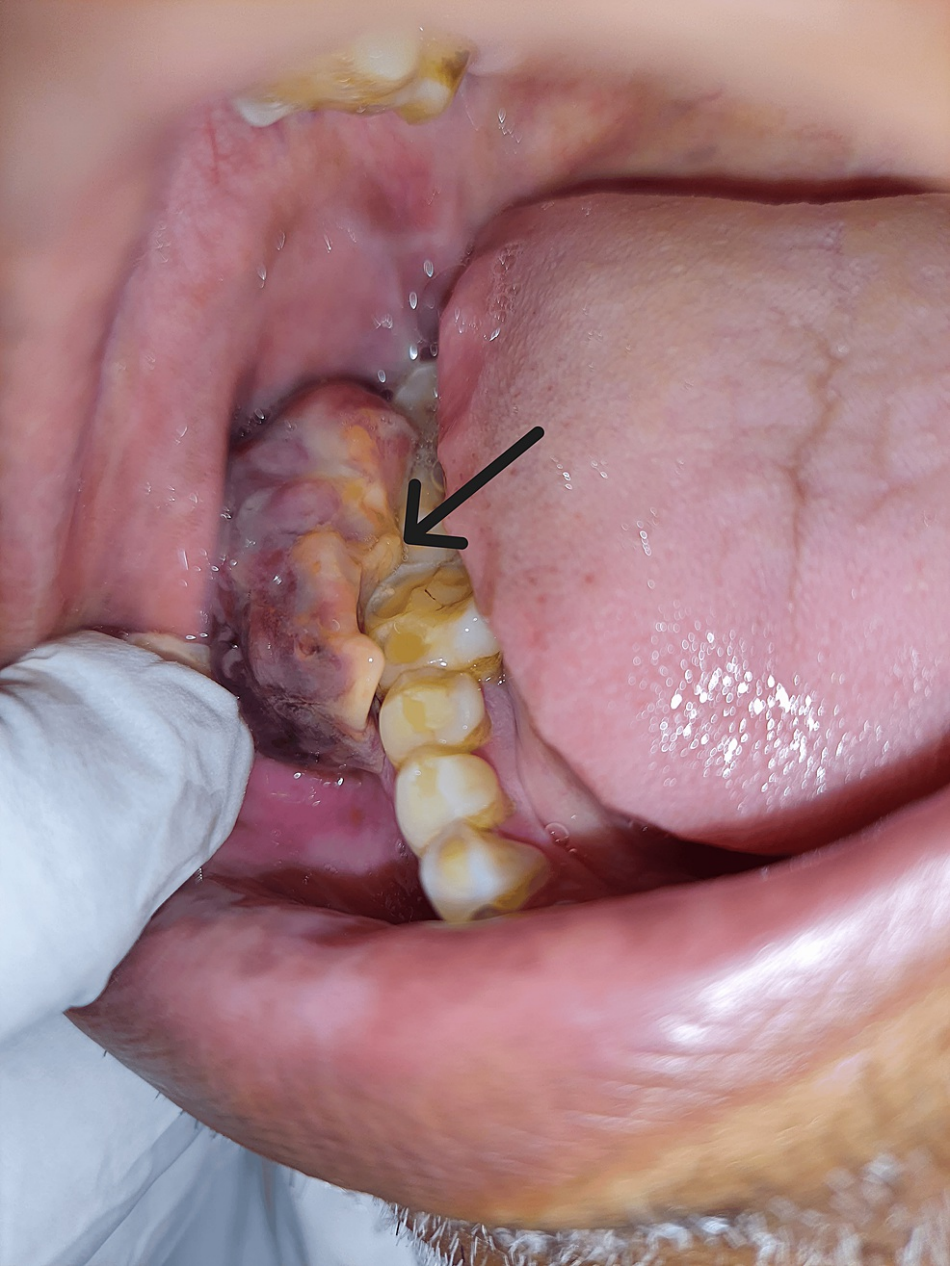
Intraoral examination showing the presence of a soft to firm, sessile, reddish pink, round to ovoid, lobulated, exophytic growth over the right gingivobuccal sulcus.

Intraorally, on inspection, we noted the presence of a reddish pink, round to ovoid, lobulated, exophytic growth over the gingivobuccal sulcus in relation to tooth numbers 46 to 48. On palpation, the growth was found to be soft to firm, sessile, roughly measuring 4 cm x 2.5 cm x 2 cm in dimensions, and was non-tender but bled on mild provocation (Figure 2).

Investigations

**Figure 3:**
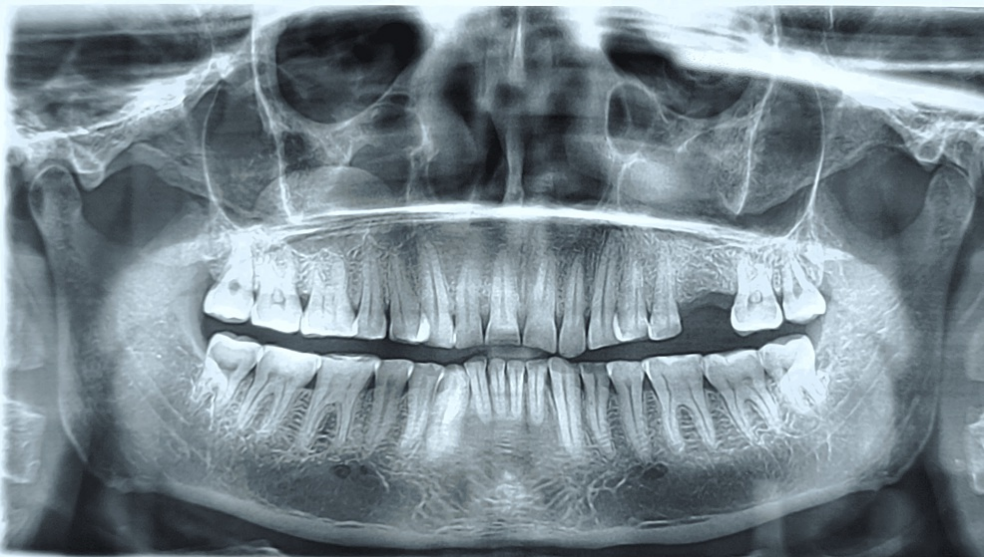
Orthopantomogram (OPG) showing no significant dental or osseous changes.

**Figure 4:**
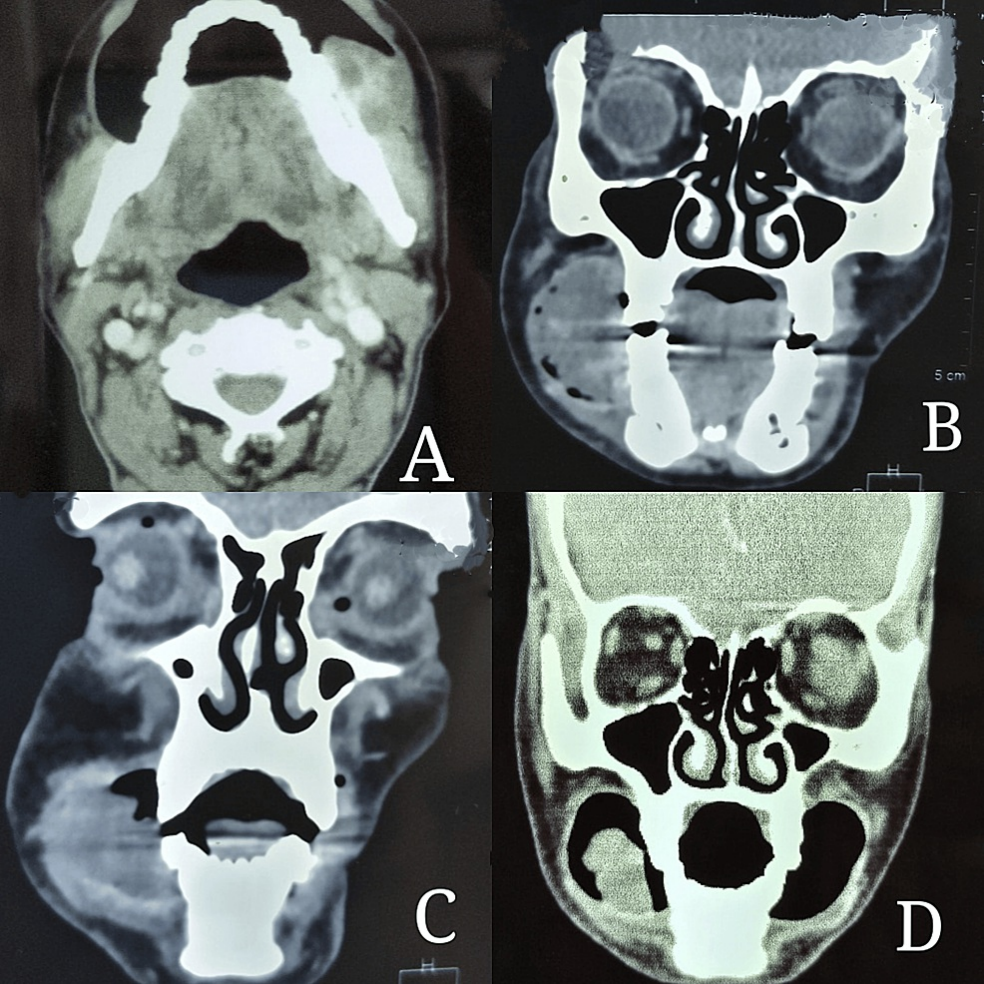
Computerized tomography (CT) scan showed an ill-defined soft tissue-attenuated lesion involving the buccal mucosa on the right side.

We failed to elicit any dental or osseous changes in OPG as can be seen in Figure 3, while computerized tomography (CT) scan showed an ill-defined soft-tissue attenuated lesion measuring 2.3 x 2.6 x 4.3 cm (in AP x TR x CC dimension, respectively) involving the buccal mucosa and extending to the upper and lower gingivobuccal sulcus on right side extending from tooth number 43 up to 48 (Figure 4). The lesion showed heterogeneous post-contrast enhancement with multiple non-enhancing areas which are suggestive of necrotic areas. No evidence of calcification was noted. The lesion was found to be causing erosion of the medial aspect of the ramus of the mandible along with the mandibular canal on the right side.

Extension of the lesion according to CT scan

Antero-laterally, it was related to the overlying subcutaneous tissue with loss of fat planes with the buccinator muscle, while posteriorly it was related to the masseter muscle with loss of fat planes. Medially, it was invading the temporalis muscle with loss of fat planes and the medial pterygoid muscle extending up to the retromolar trigone on right side. Few homogenously enhancing lymph nodes are noted at levels IA, IB, II, and III on right side.

The patient was otherwise healthy and all routine blood investigations were within normal limits, except for erythrocyte sedimentation rate (44 mm/hour) and C-reactive protein (64.87 mg/L) which were raised, and the patient tested negative for HIV I, II, and hepatitis B and C. Based on the above clinical and radiological findings, our provisional diagnosis was in favor of an aggressive malignant neoplasm.

**Figure 5:**
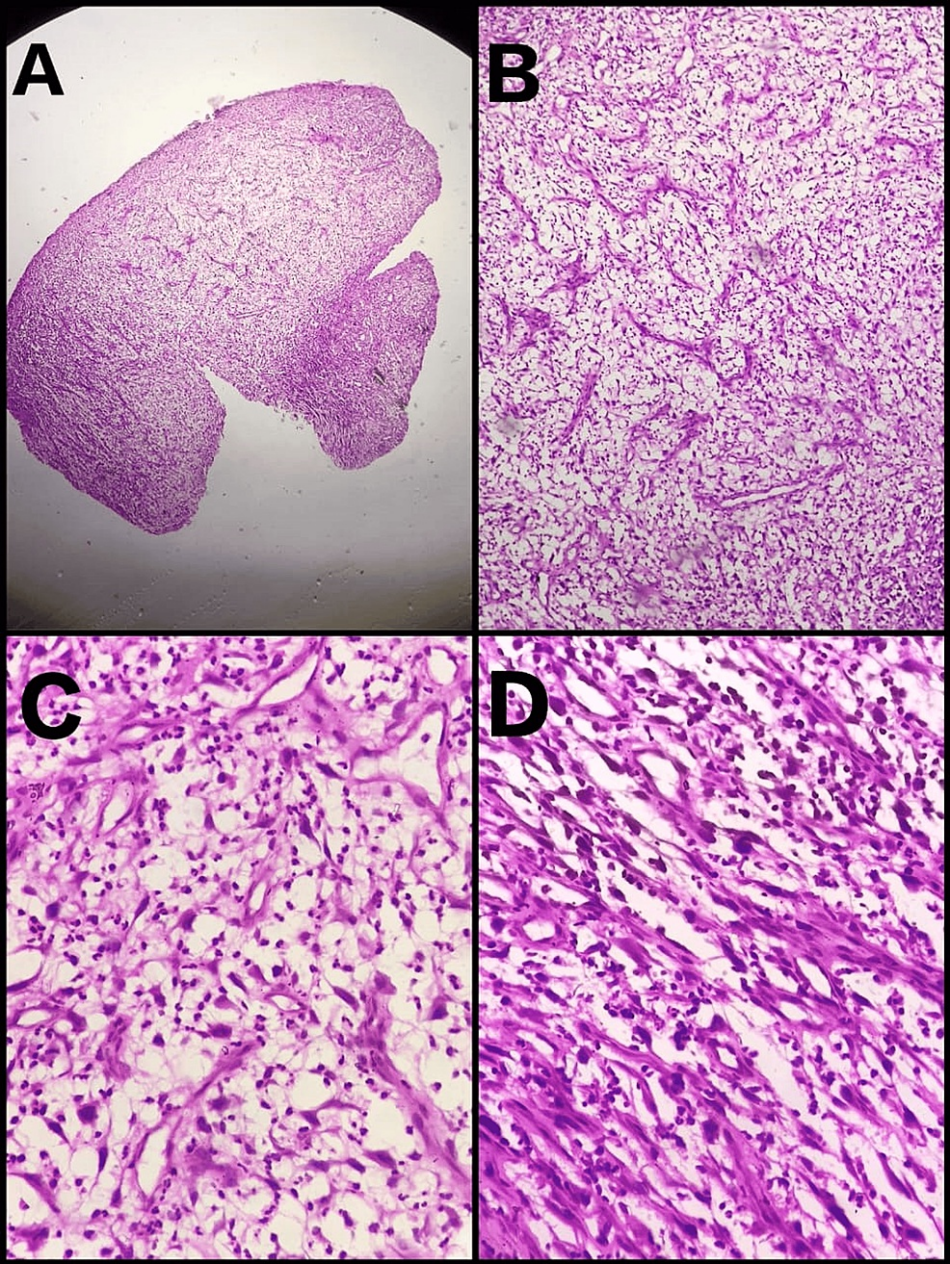
Histopathological photomicrographs in 4x (A), 10x(B), and 40x (C & D) magnification as obtained from incisional biopsy.

An incisional biopsy was performed from the proper representative site of the lesion and histopathological evaluation was done. H&E-stained sections revealed the presence of proliferative cells arranged in lobules and nests of spindled cells having clear to eosinophilic cytoplasm and hyperchromatic, pleomorphic nuclei with prominent nucleoli; abundant neutrophils and blood vessels were separating the tumor cell nests. Focal areas of necrosis and hemorrhage and brisk mitosis were noted (Figure 5).

**Figure 6:**
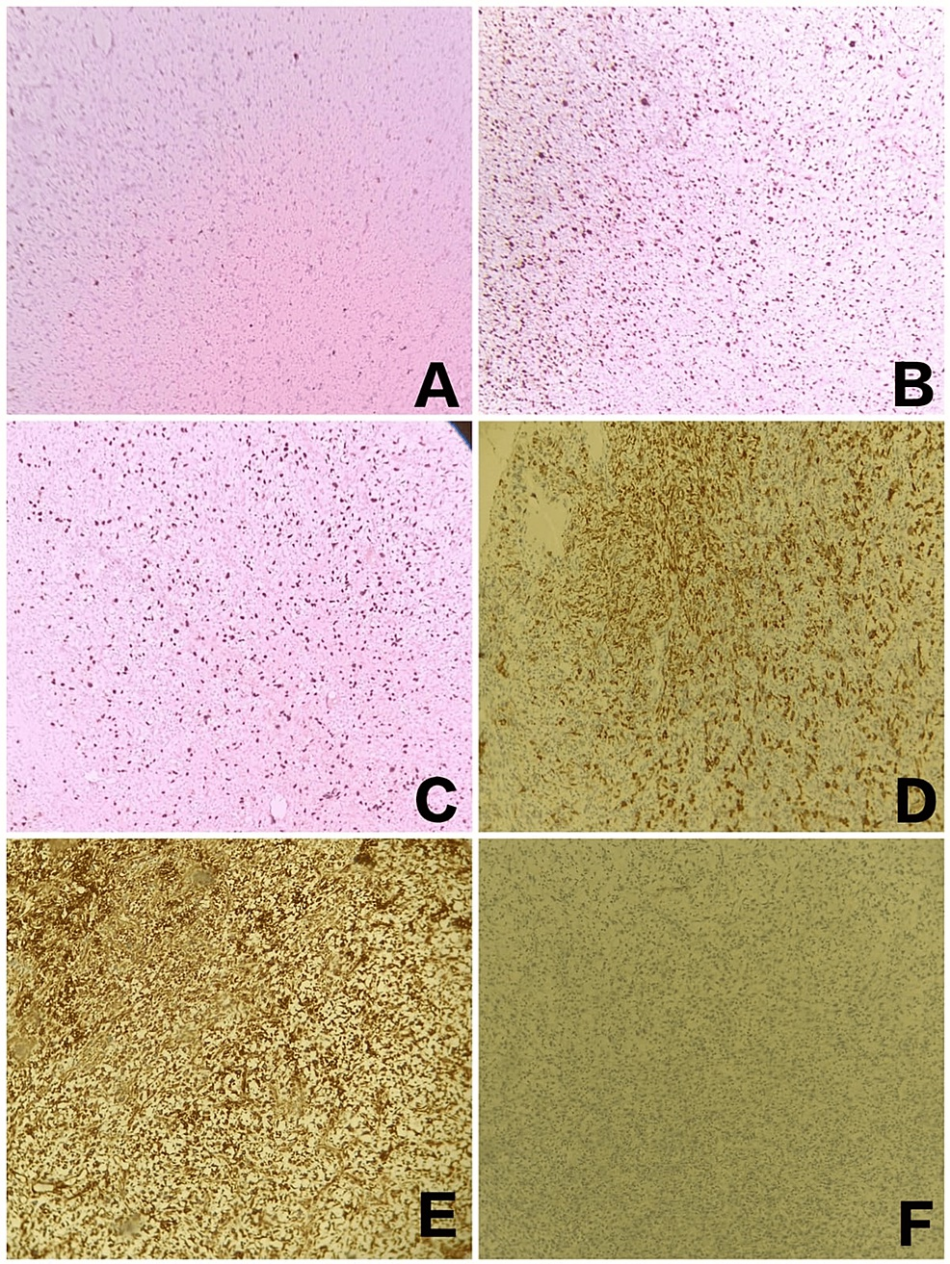
Immunohistochemistry panel depicting markers S-100 (A), p63 (B), ki67 (C), PAN CK (D), vimentin (E), desmin (F) at 10x magnification.

The overall histopathological features revealed findings suggestive of an aggressive malignant neoplasm of epithelial and/or mesenchymal origin. Immunohistochemistry was performed for confirmatory diagnosis and it showed positivity for p63 (strong and nuclear positivity), S-100 (scattered positivity), vimentin, PAN CK, p40 (partly positive), and a high ki67 index (70 to 80%). The section showed negativity for alpha-smooth muscle actin (SMA), desmin, and CD34 (Figure 6).

PAN CK positivity shows the epithelial component to be present, also vimentin positivity shows mesenchymal origin. Negativity for SMA, desmin, and CD34 rules out muscular or vascular origin of this tumor.

By amalgamation of clinical features, radiologic interpretation, hematological parameters, and histopathological evaluation, a confirmatory diagnosis of ‘Sarcomatoid Carcinoma’ was made and the patient was referred to the Department of Oral Surgery for further surgical treatment and necessary management.

**Figure 7:**
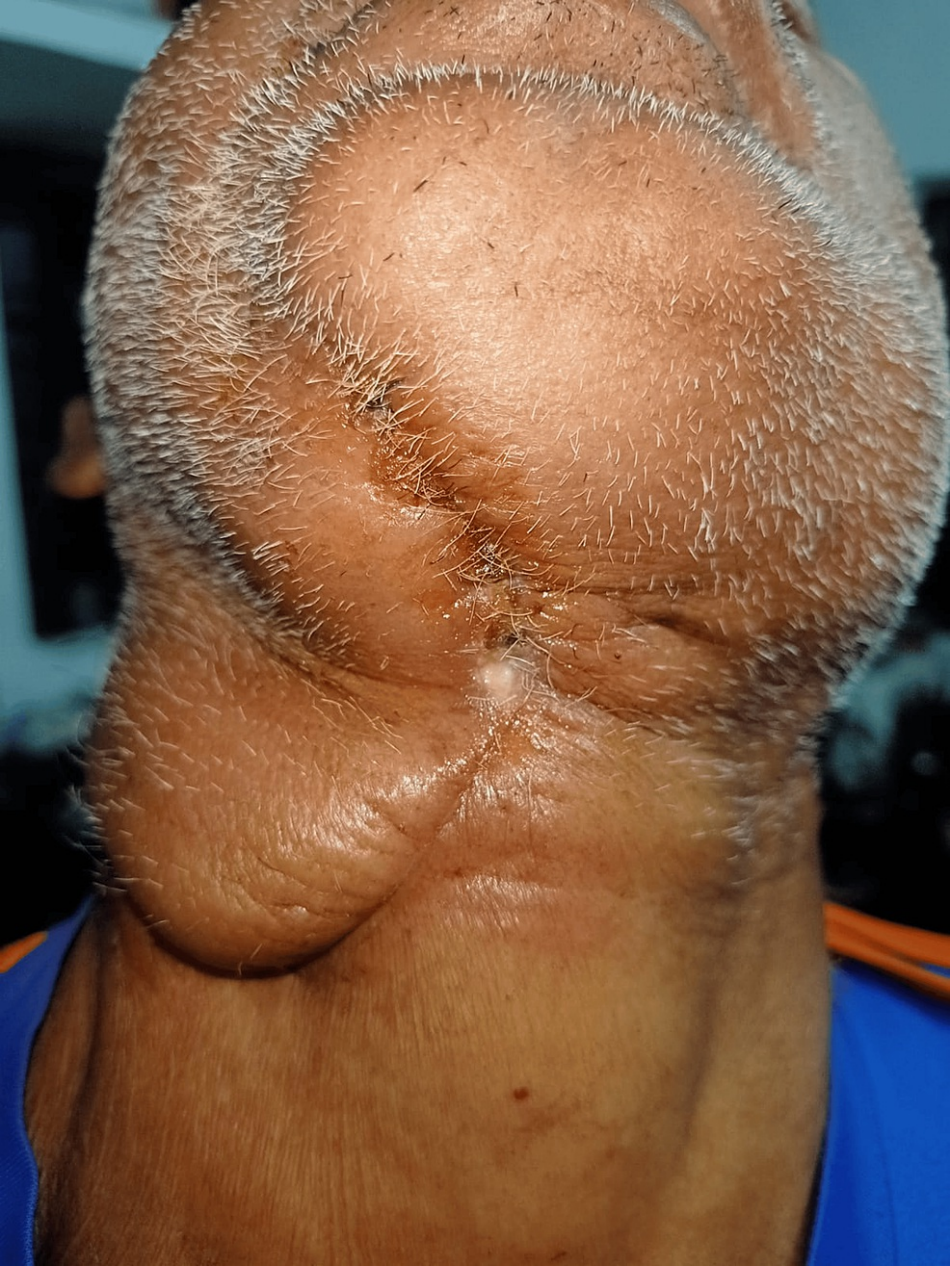
Postoperative photograph showing submandibular region after wide local excision of the tumor along with right segmental mandibulectomy and right supraomohyoid neck dissection.

The patient underwent wide local excision of the tumor along with right segmental mandibulectomy and right supraomohyoid neck dissection with removal of level I to IV lymph nodes (Figure 7).

**Figure 8:**
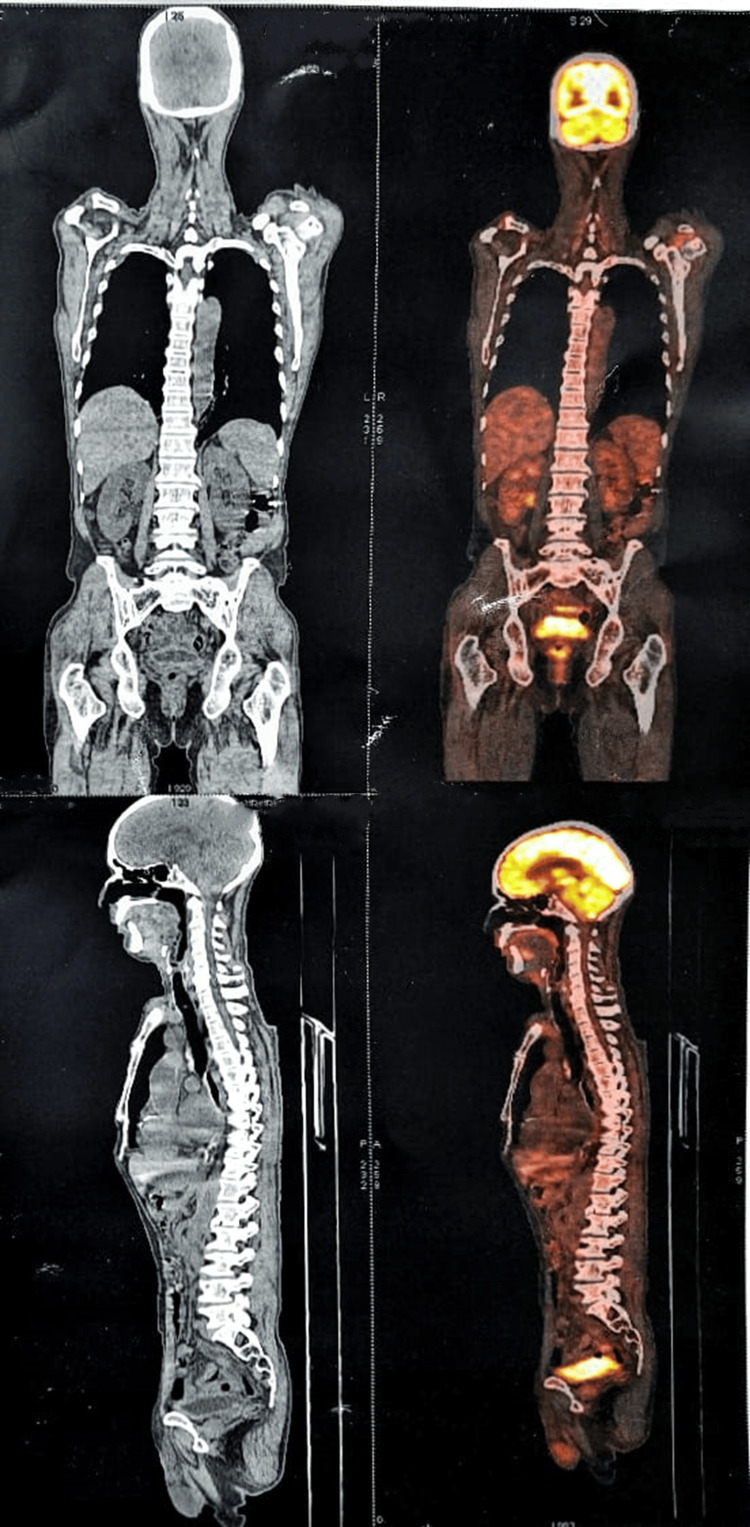
Whole-body PET-CT scan of the patient shows multiple metabolically active left metastatic cervical lymphadenopathies, with a small active lytic lesion in the right ramus of the mandible near retro-molar trigone and focally active nodular lesion in left lateral zone of the prostate. PET-CT: Positron emission tomography–computed tomography

After a month, the patient reported to our OPD with some discomfort, pain, and swelling over the left side of the lower third of the face and complained about the inability to open the mouth. We performed fine needle aspiration cytology (FNAC) from the left side level II lymph node which showed positive metastasis of tumor cells and a whole-body positron emission tomography-computed tomography (PET-CT) scan was conducted which showed multiple metabolically active left metastatic cervical lymphadenopathies. Moreover, there was a small active lytic lesion in the right ramus of the mandible near retro-molar trigone, likely local residual tissue after surgery. And focally active nodular lesion in the left lateral zone of the prostrate (Figure 8).

The patient was referred to advanced cancer center for further management and he received chemotherapeutic treatment with drugs paclitaxel 250 mg and carboplatin 450 mg, intravenous infusion, and was referred for palliative therapy as the patient was denying for second surgery and counting the days.

## Discussion

In our case, the lesion originated from the gingivobuccal sulcus, which is considered a rare site.

Oral cavity tumors usually affect the lip, alveolar ridge, and tongue and clinically the common manifestations include swelling, pain, a nonhealing ulcer, dysphagia, or hemorrhage. A distinctive clinical and pathologic feature of SpCC is its macroscopic growth pattern. Laryngeal tumors (more than 90%) and oral tumors (approximately 50%) present as polypoid and exophytic masses protruding from the lumen. Sometimes, a narrow stalk is also seen [2]. The clinical aspects of our patient were consistent with those previously reported by other writers.

Histopathologically, this tumor is typically formed by the mesenchymal component and the epithelial component often blends into it [8]. Approximately 66 to 75% of SpCC are biphasic tumors with areas of conventional SCC intertwined with areas of spindled and/or pleomorphic tumor cells with a wide variety of architectural patterns, including fascicular [2]. Our case showed similar histologic presentations.

The spindle form of tumor cells is thought to be caused by the absence of expression of cell adhesion molecules, such as cadherins, and the keratin filament network that results from this [8].

The pathogenetic conundrum has been undetermined by ultrastructural studies using electron microscopy. According to Goellner et al., the spindle cell component of these bimorphic tumors is composed of non-neoplastic histiocytes and fibroblastic cells. Minckler et al., as well as Martin et al., demonstrated that the spindle elements were malignant fibroblasts. The spindle cells appeared mesenchymal but were epithelial in origin, according to Leifer et al., Battifora discovered that epithelial cells converted into mesenchymal cells [7].

Abundant aberrant mitotic figures can be simply described and rarely, metaplastic, or frank neoplastic cartilage or bone can be noticed. In most cases, surface squamous neoplasia is unremarkable due to the extensive surface ulceration, which intensifies the need for immunohistochemistry as a prerequisite for SpCC diagnosis [1].

Immunohistochemical studies are beneficial to understanding the histogenesis of the spindle cells within these tumors and the nature of SpCC. There are multiple histogenetic hypotheses have been demonstrated over the years, with 3 principal theories, including, firstly, it is a collision tumor or a carcinosarcoma; whereby a separate epithelial and mesenchymal cell have each become malignant. Second, it is a SpCC or sarcomatoid carcinoma - an epithelial cell that differentiates into spindle cell components; or it can be a pseudosarcoma - an SpCC that undergoes benign reactive stromal [6].

AE1/AE3, pan-cytokeratin are the most commonly used epithelial stains. Epithelial membrane antigen (EMA), and p63. When combined, 65%-97% of SpCC will respond to at least one of the epithelial markers. In contrast, 100% of SpCC cases show positivity for vimentin but high variability to other mesenchymal markers such as smooth muscle actin and muscle-specific actin [6].

In our case, immunohistochemistry was performed for confirmatory diagnosis and it showed positivity for p63 (strong and nuclear positivity), S-100 (scattered positivity), vimentin, PAN CK, p40 (partly positive), and a high ki67 index (70 to 80%) and negativity for SMA, desmin, CD34. SC is potentially aggressive and appears to return and metastasize readily. It should be treated as such, with superior therapy aiming to limit both local and distant recurrence. Surgery is the most well-established mode of first final therapy for the vast majority of oral malignancies; its objective is to eradicate the cancer, maintain or restore form and function, reduce treatment adverse effects, and eventually prevent any new primary malignancies. Surgery is employed in the majority of instances due to the convenience of treatment and excellent results in terms of cure and postoperative function [9].

Some authors believe that wide radical resection is sufficient on its own, whereas others believe that surgery combined with radiotherapy is preferable. Surgery, followed by radiotherapy, provides the greatest long-term outcome for the patient [6].

In the presented case here, the treatment modality followed was majorly surgical with wide local excision of the tumor along with right segmental mandibulectomy and right supraomohyoid neck dissection with removal of level I to IV lymph nodes followed by chemotherapy.

## Conclusions

SpCC is a rare and unusual variant of squamous cell carcinoma which is often difficult to diagnose due to its biphasic nature consisting of cells of both epithelial and mesenchymal origin. Apart from its evident diagnostic difficulty which is best resolved via proper histological and immunohistological evaluation, there is an added challenge to the clinician due to its unusually high metastatic potential. All these criteria make the disease very aggressive which requires quick diagnosis and dire treatment plans to reduce the spread and morbidity of the disease.
